# A Search Engine to Access PubMed Monolingual Subsets: Proof of Concept and Evaluation in French

**DOI:** 10.2196/jmir.3836

**Published:** 2014-12-01

**Authors:** Nicolas Griffon, Matthieu Schuers, Lina Fatima Soualmia, Julien Grosjean, Gaétan Kerdelhué, Ivan Kergourlay, Badisse Dahamna, Stéfan Jacques Darmoni

**Affiliations:** ^1^CISMeFTIBS, LITIS EA 4108Rouen University Hospital, NormandyRouenFrance; ^2^U1142, LIMICSInsermParisFrance; ^3^UMR_S 1142, LIMICSUPMC Univ Paris 06Sorbonne UniversitésParisFrance; ^4^LIMICS (UMR_S 1142)Univ. Paris 13Sorbonne Paris CitéVilletaneuseFrance; ^5^Département de Médecine GénéraleUniversité de RouenRouenFrance

**Keywords:** databases, bibliographic, French language, information storage and retrieval, PubMed, user-computer interface, search engine

## Abstract

**Background:**

PubMed contains numerous articles in languages other than English. However, existing solutions to access these articles in the language in which they were written remain unconvincing.

**Objective:**

The aim of this study was to propose a practical search engine, called Multilingual PubMed, which will permit access to a PubMed subset in 1 language and to evaluate the precision and coverage for the French version (Multilingual PubMed-French).

**Methods:**

To create this tool, translations of MeSH were enriched (eg, adding synonyms and translations in French) and integrated into a terminology portal. PubMed subsets in several European languages were also added to our database using a dedicated parser. The response time for the generic semantic search engine was evaluated for simple queries. BabelMeSH, Multilingual PubMed-French, and 3 different PubMed strategies were compared by searching for literature in French. Precision and coverage were measured for 20 randomly selected queries. The results were evaluated as relevant to title and abstract, the evaluator being blind to search strategy.

**Results:**

More than 650,000 PubMed citations in French were integrated into the Multilingual PubMed-French information system. The response times were all below the threshold defined for usability (2 seconds). Two search strategies (Multilingual PubMed-French and 1 PubMed strategy) showed high precision (0.93 and 0.97, respectively), but coverage was 4 times higher for Multilingual PubMed-French.

**Conclusions:**

It is now possible to freely access biomedical literature using a practical search tool in French. This tool will be of particular interest for health professionals and other end users who do not read or query sufficiently in English. The information system is theoretically well suited to expand the approach to other European languages, such as German, Spanish, Norwegian, and Portuguese.

## Introduction

MEDLINE, created by the US National Library of Medicine (NLM), is the most used medical bibliographic database in the world. Currently (as of September 3, 2014), it contains 21,515,657 citations [1] from 5650 indexed journals from 81 countries around the world. Each MEDLINE record is indexed with the NLM’s controlled vocabulary, Medical Subject Headings (MeSH) [2].

MEDLINE is the largest component of PubMed [3], the freely accessible online database of biomedical journal citations and abstracts. In addition to MEDLINE citations, PubMed also contains [4]:

1. Citations not yet indexed with MeSH and added to MEDLINE (in-process citations or when supplied electronically by the publisher);
2. Some “old MEDLINE” citations that have not yet been updated with the current vocabulary and converted to MEDLINE status;
3. Citations to some additional life science journals that submit full text to PubMedCentral and receive a qualitative review by the NLM; and
4. Citations to author manuscripts of articles published by NIH-funded researchers.

On the same date (September 3, 2014), PubMed contained 24,157,837 citations [5].

Language may be an obstacle to access PubMed and all the information it contains [6]. Moreover, there is still demand for native-language articles [7]. Of the 28 different ways to access PubMed listed by Lu [8], only 2 help non-native-English speakers to query PubMed/MEDLINE in their native language: BabelMeSH [9,10] and Patient, Intervention, Comparison, Outcome (PICO) Linguist [10]. We developed a French MeSH browser linked to PubMed [11]; more than 500 users consult it daily and it is taught in half of French medical schools. These 3 tools rely on MeSH translation in multiple languages to ease querying [12] and also rely on some metadata available in existing user languages to ease the results of browsing. Nevertheless, these 3 tools lack most PubMed functionalities (eg, advanced query builder, filters, citation sensor); in truth, they just lack functionalities.

A multilingual search engine to access the PubMed/MEDLINE subset in any non-English language (eg, French, German, Spanish, or Norwegian) with advanced functionalities would be of great interest for any user who is not comfortable in English. The goal of this paper was to present such a tool (Multilingual PubMed) and evaluate the potential increase in information retrieval task performance by using the French version.

## Methods

### Material

To create an effective multilingual search engine, the basic framework must first be clearly specified. Several tools were initially developed before the beginning of this project. Since 1995, the Catalogue and Index of Online Health Resources in French (CISMeF=Catalogue et Index des Sites Médicaux de langue Française), a quality-controlled health gateway [13], describes and indexes using the MeSH thesaurus the main institutional health Web resources (documents and sites) available in French. A search engine, Doc’CISMeF, was developed in 2000 to allow querying of this gateway. In 2007, CISMeF began using several terminologies/ontologies to allow easier and more accurate indexing. The CISMeF team was prompted to develop a multiterminology portal (French/English) [14], which interoperated with the search engine. In 2012, a transition was made to establish a cross-lingual portal named Health Terminology/Ontology Portal (HeTOP) available in 23 different languages [15,16].

Recently, Doc’CISMeF was significantly updated in 2 ways: (1) it has become a generic tool able to integrate any metadata model and its related data; therefore, this tool is able to describe and index Web resources as well as PubMed citations and (2) the search engine has now been developed into a reliable multilingual tool, which is capable of searching by using multiple terminologies/ontologies in several languages.

These 2 improvements in Doc’CISMeF allow the search for PubMed citation in any language. The final steps to transform Doc’CISMeF into the desired Multilingual Access to Each PubMed Subset for Each Language (Multilingual PubMed or, for French, Multilingual PubMed-French), designed for non-English speakers to access the literature in languages other than English, consist of (1) completing translation of MeSH descriptors and MeSH qualifiers and (2) translating the interface.

For the 5 basic languages of this study, the translation of MeSH descriptors is available in Unified Medical Language System [17]. Several institutions have helped us to translate the remaining MeSH qualifiers and interface terms (see Acknowledgments).

Nevertheless, developing a search engine requires good knowledge of the language used. As a French team, we are unable to assume that the performance for all other languages is equivalent to that of French language.

Two improvements related to the MeSH thesaurus in Doc’CISMeF can be used in Multilingual PubMed [18]:

1. Bilingual (French and English) predefined queries (n=389) were defined by the CISMeF medical librarians (eg, the CISMeF term “natremia” was automatically transformed to the MeSH predefined query “sodium/blood”). These predefined queries will have to be translated to be applied to the other European languages of this project.
2. Superconcepts (or metaterms; n=126) are medical (sub)specialties or biological science disciplines (eg, cardiology or bacteriology) selected by the CISMeF chief librarian. For each superconcept, 1 semantic link was created with 1 or more MeSH descriptor and qualifier. For example, the metaterm *psychiatry* is associated with the MeSH descriptors *psychiatry* and *psychiatric hospital* that belong to a completely different tree structure within the MeSH. Superconcepts have been created to optimize information retrieval and to overcome the relatively restrictive nature of MeSH descriptors. These superconcepts also need to be translated, but the basic semantic links between superconcepts and MeSH descriptors are language-independent.

The list of predefined queries and metaterms is available at the HeTOP cross-lingual terminologies/ontologies portal [15].

There are also some other improvements that are based on all the available metadata. It is possible to perform faceted browsing [19]. Facets list citation characteristics, which can be used to refine results. It works based on metadata (eg, year, country, resource type). Default ranking in Doc’CISMeF is based on a relevance score, whereas in PubMed, the most recent articles are ranked first: last in-first out (LIFO). Doc’CISMeF interprets users’ queries to identify meaningful words and medical descriptors matching health terminologies, such as MeSH. It then returns results corresponding to these words and descriptors. It uses several criteria to score and rank the results: query words present in the title, descriptors present in major topics, publication date, and origin of the descriptors as they may have been assigned by librarians or programmatically by computers for less important resources. If title words and major topics perfectly matched the user’s query, they score 100% and are consequently ranked. Imperfect matches score less depending on how many words and descriptors are present (eg, MeSH minor topic). For results of equal scores, publication date is the determinant criterion.

Some other improvements previously developed in Doc’CISMeF have not yet been implemented in the Multilingual PubMed-French search engine (see Discussion).

### Architecture

**Figure 1:**
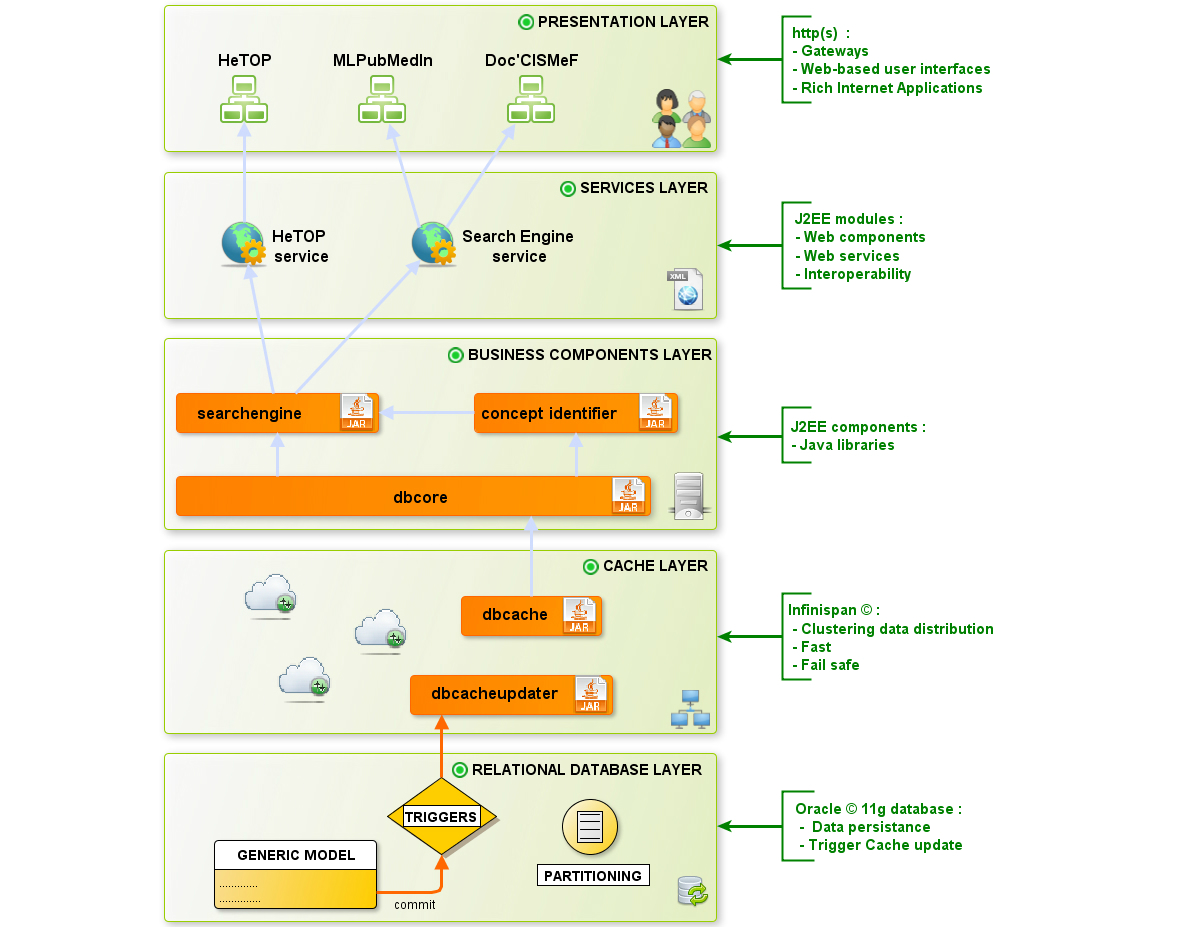
CISMeF information system.

The CISMeF generic information system is divided into 5 layers (Figure 1):

1. A relational database layer, which ensures data persistence and Structured Query Language query processing; execution plans are optimized by dynamic partitioning and local indexes.
2. A cache layer, which is devoted to clustering data distribution. It is based on random access memory and is distributed between several servers; therefore, it is very fast. Moreover, it avoids data loss after crashes.
3. A business components layer mainly composed of CISMeF Java libraries dealing with business logic.
4. A service layer hosting Web services that rely on business components and are, in turn, used by each client application of the information system.
5. A presentation layer that includes the gateways, Web-based user interfaces, rich Internet apps, and any client application of the service layer.

### Data Sources

The first and only version of Multilingual PubMed tested in this paper was the French version: the Multilingual PubMed-French search engine. Nevertheless, the whole framework should be able to manage any language as it does with French. To perform this study, all PubMed citations in French were extracted from PubMed and included in the Multilingual PubMed-French semantic search engine using a specific parser. Some PubMed citations were also extracted in other languages (ie, German, Spanish, Portuguese, and Norwegian) to ensure that our hypothesis of a multilingual framework was not unrealistic.

All MeSH descriptors and MeSH qualifiers were translated into French by the Technical and Scientific Information Department of the French National Institute of Health [20]. The CISMeF team considerably enriched this translation with:

1. 25,501 synonyms and 689 ambiguous acronyms of MeSH descriptors;
2. 163 synonyms of MeSH qualifiers;
3. 20,887 translations of MeSH supplementary concepts (out of 209,326);
4. 27,295 synonyms of MeSH supplementary concepts;
5. 6037 translations of MeSH scope notes, which consist of MeSH descriptor definitions; and
6. 3918 “see also” relations among MeSH descriptors.

### Evaluation

The 2 main limiting factors for such a tool are the response time, which must be low in order to be acceptable for end users, and the result quality (are there any results and are they relevant?). They were evaluated by measuring the response time and relevance of the first 20 results for 20 queries.

Queries were selected from the queries frequently run on Doc’CISMeF (log-analysis). Twenty queries were randomly selected from those that retrieved results, were run more than 50 times in the previous 100 days, and did not involve advanced syntax.

Each query was run on Multilingual PubMed-French, BabelMeSH, and PubMed; the exact sentence from our log was pasted in each search engine. The corpus was limited to articles only in French language for each search engine. For PubMed, a second query was launched on the transliterated title (TT) field, hereafter referred to as the PubMed TT strategy. Finally, 2 PubMed algorithms for result sorting were tested: the classic LIFO and the recently proposed relevance ranking [21], which is probably more comparable to the Multilingual PubMed-French sorting algorithm. Thus, 5 strategies were tested: BabelMeSH, Multilingual PubMed-French, PubMed LIFO, PubMed relevance, and PubMed TT. Examples of queries are available in Table 1. PICO linguist was not evaluated because it works basically like Babel MeSH, but with a PICO interface (ie, with structured fields for patient, intervention, comparison, and outcome) which is designed for a specific query. The PubMed TT strategy was only studied with LIFO ranking because relevance ranking is based on the field in which the terms are found [21] and the PubMed TT strategy involved only 1 field: transliterated title. According to our data, PubMed LIFO and PubMed relevance ranking share 45% of their results. For PubMed TT strategy, this proportion rose to 88%.

The PubMed identifier (PMID) of the first 20 citations and the total number of citations were recorded by NG. For Multilingual PubMed-French, only response times were recorded. A medical librarian (GK) performed an assessment of citation relevance using a 3-modality Likert scale. He was blind to the search strategy that retrieved the resources to be evaluated. Therefore, it is possible to estimate the precision of each tool (ie, the proportion of relevant results in the results).

The relation between response time and number of citations was studied using the Pearson correlation coefficient. Two analyses were performed: for the strict one, only citations assessed as totally relevant were considered as true positives; for the relaxed one, both totally relevant and partially relevant citations were considered as true positives.

**Table 1:** Examples of queries in the different tools.

Search strategy	Example of query (for “constipation”)^a^
BabelMeSH^b^	constipation
Multilingual PubMed-French	constipation
PubMed^c^	constipation[All Fields] AND French[lang]
PubMed TT	constipation[TT] AND French[lang]

^a^ Shown is the query as written in the search box.

^b^ The checkbox “français” was checked.

^c^ Whatever the ranking used (relevance or LIFO), the query was the same.

## Results

### Technical Feasibility

**Figure 2:**
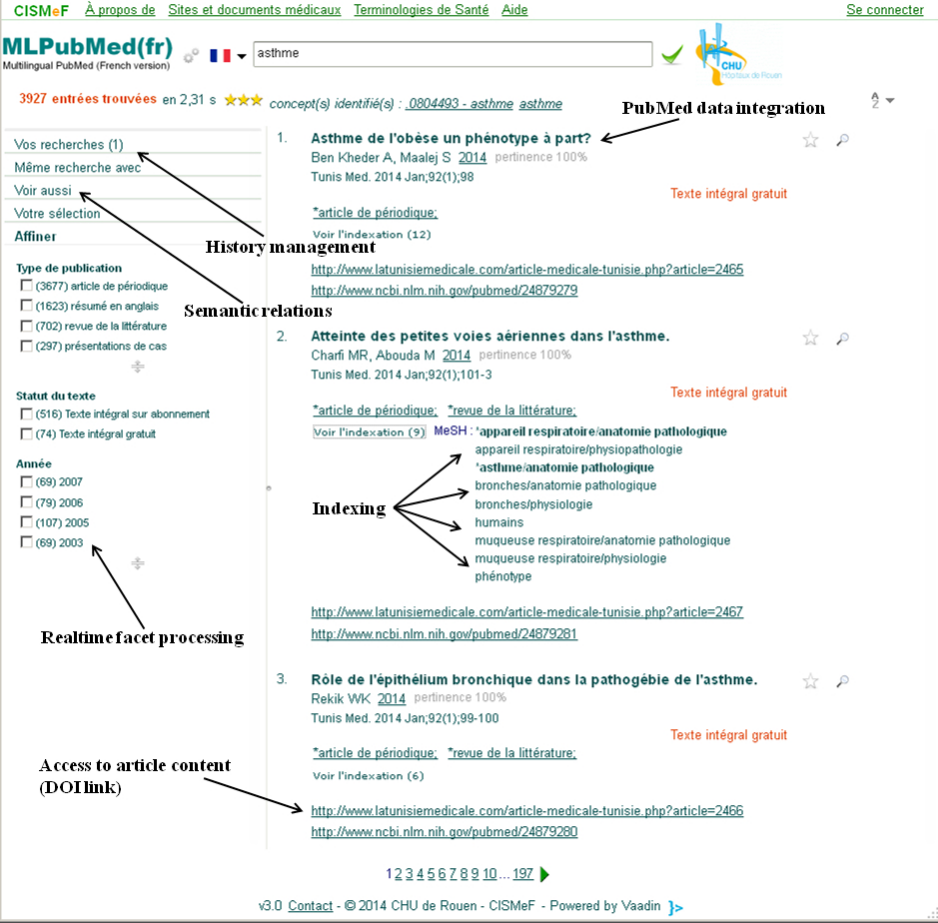
Screenshot of the multilingual search engine.

Table 2 shows the number of PubMed citations for the main European languages (excluding English). As a proof of concept, PubMed citations of different languages were included in the CISMeF information system: French (n=665,359), German (n=7102), Portuguese (n=4497), Spanish (n=4297), and Norwegian (n=3764). The end user was able to choose 1 language and then perform a query in the same language. Thanks to our colleagues mentioned in the Acknowledgments, the Multilingual PubMed interface was translated into French, German, Portuguese, Spanish, and Norwegian. The same main metadata displayed by default in the PubMed database was similarly displayed in the Multilingual PubMed search engine, as well as the indexing MeSH descriptors and qualifiers (Figure 2). Direct links to the full-text article in the same language were also presented to the end user (Figure 2), often via Digital Object Identifier [22]. The objective of creating a bibliographic database extracted from PubMed in several languages and available in 1 specific language was then completed as a proof of concept.

In the previous CISMeF gateway, the objective for response time was the following: all queries with 1 or 2 terms should be less than 2 seconds. Since 2001, this objective has been fulfilled for the 101,000 Web resources included in the CISMeF information system. The number of PubMed citations in French versus CISMeF Web resources is approximately 6 times more. All response times for all queries were below the limit of 2 seconds (Table 3). As expected, there was a strong correlation between the response time and the number of citations retrieved (*r*=.73, 95% CI .42-.89). According to linear regression coefficient, this would lead to an unacceptable response time for queries that collect more than 20,000 citations. This evaluation of response time is an important step in determining the feasibility of this Multilingual PubMed-French search engine to create a bibliographic database in French for health professionals. The current version of Multilingual PubMed-French is available on the Internet [23].

**Table 2:** Number of PubMed citations for the main European languages.

Language	Number of PubMed citations
German	808,653
French	680,451^a^
Italian	294,720
Spanish	302,287
Portuguese	85,839
Norwegian	35,712

^a^ The discrepancy between the number of French citations in PubMed and the number of French citations inserted in our information system comes primarily from some doubtful classifications in PubMed (ie, articles mislabeled as French).

**Table 3:** Response times for the 20 Multilingual PubMed-French queries.

Query (French/English)	Response time (seconds)	Number of PubMed citations
Allaitement maternel / breast feeding	0.42	633
Angine/pharyngitis	0.53	845
AVC/stroke	0.54	1704
BPCO/COPD	0.49	1503
Cigarette électronique / electronic cigarette	0.42	24
Constipation/constipation	0.27	531
Coqueluche / whooping cough	0.54	314
Gale/scabies	0.57	148
Hémochromatose/hemochromatosis	0.59	712
Hypertension/hypertension	1.03	8694
Lupus/lupus	0.34	1147
Maladie cœliaque / celiac disease	0.58	723
Maladie de Crohn / Crohn disease	0.69	1363
Nutrition/nutrition	1.13	8367
Psychiatrie/psychiatry	0.94	5602
Sarcoïdose/sarcoidosis	0.77	1721
Scoliose/scoliosis	1.10	669
Soins infirmiers / nursing care	0.86	5767
Tabac/tobacco	0.42	679
Toxoplasmose/toxoplasmosis	0.74	1238

### Performance Evaluation

Table 4 displays the coverage of each query for each strategy. The PubMed TT strategy retrieved far fewer citations than the other 4 strategies with a total of 10,716 hits. Babel MeSH, Multilingual PubMed-French, and regular PubMed queries retrieved 50,894, 42,384, and 34,047 citations, respectively. Babel MeSH and Multilingual PubMed-French retrieved significantly more citations than relevance/LIFO PubMed (both *P*=.03; Mann-Whitney test).

The precision measured on the first 20 citations retrieved are listed (1) considering the citations assessed as highly relevant—the strict analysis—and (2) considering both the highly and the slightly relevant citations, the relaxed analysis (Table 5). The results were similar with 2 strategies that outperformed the other 3 in terms of precision: Multilingual PubMed-French and PubMed TT. These 2 strategies reached more than 90% precision with a slight advantage for PubMed TT (*P*=.002, Fisher test), whereas the other 3 barely reached 80% in the relaxed analysis.

**Table 4:** Coverage of queries according to the search engine.

Query (English)	Strategy (n)			
	BabelMeSH	Multilingual PubMed-French	PubMed^a^	PubMed TT
Breast feeding	639	633	3	50
Pharyngitis	2043	845	2326	82
Stroke	2671	1704	202	52
COPD	687	1503	25	275
Electronic cigarette	355	24	0	0
Constipation	574	531	666	144
Whooping cough	227	314	2	166
Scabies	136	148	4	102
Hemochromatosis	537	712	756	129
Hypertension	10,348	8694	12,580	1533
Lupus	2232	1147	2694	1150
Celiac disease	649	723	2	305
Crohn disease	1190	1363	2	758
Nutrition	4969	8367	6480	819
Psychiatry	4453	5602	5394	1940
Sarcoidosis	1522	1721	13	878
Scoliosis	651	669	3	159
Nursing care	14,360	5767	2867	888
Tobacco	1607	679	18	585
Toxoplasmosis	1044	1238	10	701
Total	50,894	42,384	34,047	10,716

^a^ Whatever the ranking used (relevance or LIFO), the number of citations retrieved was the same.

**Table 5:** Precision measured on the first 20 citations for each query for each strategy (analysis-relaxed analysis).

Query (English)	Strategy (precision)				
	Babel MeSH	Multilingual PubMed-French	PubMed LIFO	PubMed Relevance	PubMed TT
Breast feeding	0.55-0.75	1.00-1.00	1.00-1.00^a^	1.00-1.00^a^	1.00-1.00
Pharyngitis	0.00-0.00	0.65-0.70	0.00-0.00	0.00-0.00	0.50-0.60
Stroke	0.45-0.80	0.95-0.95	0.15-0.15	0.35-0.35	1.00-1.00
COPD	0.40-0.55	1.00-1.00	0.75-0.85	1.00-1.00	1.00-1.00
Electronic cigarette	0.10-0.10	0.35-0.40	—^b^	—^b^	—^b^
Constipation	0.45-1.00	1.00-1.00	0.50-0.95	0.75-1.00	1.00-1.00
Whooping cough	0.80-1.00	1.00-1.00	1.00-1.00^c^	1.00-1.00^c^	1.00-1.00
Scabies	0.90-1.00	1.00-1.00	0.25-0.25^d^	0.25-0.25^d^	1.00-1.00
Hemochromatosis	0.65-0.95	1.00-1.00	0.70-1.00	0.85-1.00	1.00-1.00
Hypertension	0.45-0.75	0.95-0.95	0.25-0.75	1.00-1.00	1.00-1.00
Lupus	0.70-1.00	1.00-1.00	0.75-0.95	1.00-1.00	1.00-1.00
Celiac disease	0.70-0.95	1.00-1.00	1.00-1.00^c^	1.00-1.00^c^	1.00-1.00
Crohn disease	0.80-0.90	1.00-1.00	0.50-1.00^c^	0.50-1.00^c^	1.00-1.00
Nutrition	0.45-0.70	0.70-1.00	0.25-0.60	1.00-1.00	1.00-1.00
Psychiatry	0.75-0.80	1.00-1.00	0.85-0.95	0.90-0.95	1.00-1.00
Sarcoidosis	0.70-0.90	1.00-1.00	0.92-0.92^e^	0.92-0.92^e^	1.00-1.00
Scoliosis	0.65-0.90	1.00-1.00	0.67-0.67^a^	0.67-0.67^a^	1.00-1.00
Nursing care	0.85-0.90	1.00-1.00	0.70-0.80	0.85-0.90	1.00-1.00
Tobacco	0.60-0.90	1.00-1.00	0.83-0.89^f^	0.83-0.89^f^	1.00-1.00
Toxoplasmosis	0.65-0.90	1.00-1.00	1.00-1.00^g^	1.00-1.00^g^	1.00-1.00
Total	0.58-0.79	0.93-0.95	0.57-0.74	0.79-0.83	0.97-0.98

^a^ Only 3 citations retrieved.

^b^ No citation retrieved.

^c^ Only 2 citations retrieved.

^d^ Only 4 citations retrieved.

^e^ Only 13 citations retrieved.

^f^ Only 18 citations retrieved.

^g^ Only 10 citations retrieved.

## Discussion

### Principal Results

The Multilingual PubMed-French strategy allows the retrieval of French PubMed citations with high precision and high coverage. Despite more than 600,000 PubMed citations that were introduced into the CISMeF information system, the average server-side response time stayed below the threshold time (ie, 2 seconds), which is acceptable, in our experience, for the average health professional. The Multilingual PubMed-French search engine is now freely available on the Internet [23].

Babel MeSH allows users to access PubMed database in French. It provides many results for low precision and is, therefore, of poor interest for French speakers. Moreover, the functioning of the search engine is not detailed anywhere; it is only possible to make rough free-term queries that are translated automatically by Babel MeSH. The Babel MeSH interface is limited; it is not possible to perform many queries in a short period of time.

The classic use of PubMed is not satisfying for French speakers, which is not surprising because it is supposed to work in English, not in French. The relatively high mean coverage masked enormous heterogeneity (from 0 to more than 12,000 citations). Precision was lower than that for Multilingual PubMed-French or PubMed TT. However, the relevance ranking provided consistently better precision for the first 20 results than LIFO ranking. There was no evidence to maintain LIFO ranking as default.

Using PubMed TT provides more precise results than using Multilingual PubMed-French. PubMed TT works like a free-text search engine that looks only in the title of the citation. Therefore, it is quite logical that precision was high. Nevertheless, PubMed TT recall must be lower than Multilingual PubMed-French recall based on the huge differences in coverage and slight differences in precision. Moreover, Multilingual PubMed-French and Babel MeSH constantly provided more than 20 results, whereas PubMed did not; some queries provided few or even no results. The poor coverage of PubMed TT is probably the consequence of difficulties managing French particularities (eg, elision, apostrophe, accentuation).

### Limitations

This study has several limitations. First, this feasibility study only included server-side response time. The overall response time also depended on the end user’s computer type and type of browser. Second, the MeSH thesaurus is incompletely translated. MeSH descriptors and qualifiers are frequently translated, but this is not the case for MeSH supplementary concepts. Therefore, querying at a MeSH supplementary concept level of accuracy is currently not possible in one’s own language. It would require a huge amount of work to translate all MeSH supplementary concepts, even using natural language processing tools [24]. Last, English is by far the most frequently used language of scientific medical publications and publications in other languages are declining comparatively. Nevertheless, the latter are important because they can be more easily adapted to national contexts and be more understandable to a wider local audience. An automatic intelligible translation of English articles would definitely resolve this issue, but it is far beyond current technology [25].

For the “angine/pharyngitis” query, an unanticipated issue arose: PubMed (LIFO and relevance strategies) did not find any results and automatically translated the query to “angina” which has a different meaning. This affected the precision of these strategies by biasing it toward zero. Three sensitivity analyses were performed to evaluate bias importance: (1) the “angine” query was excluded from the analysis, (2) the “angine” query was considered as providing zero results for PubMed LIFO and PubMed relevance strategies, and (3) the 20 citations returned by this query for PubMed LIFO/relevance strategies were considered as fully relevant. In each scenario, the precision was significantly better for Multilingual PubMed-French and PubMed TT compared with PubMed LIFO/relevance strategies. Therefore, it is possible to conclude a very limited bias.

### Perspectives and Future Challenges

One of the strengths of Multilingual PubMed lies in the multilingual CISMeF information system. Because the MeSH thesaurus is already translated into multiple languages, it will be easy to make Multilingual PubMed for these languages. Nevertheless, the CISMeF team is not familiar with the linguistic treatments that are necessary to interpret user queries in other languages than French; therefore, it is unlikely that the other versions of Multilingual PubMed will achieve results comparable to those observed here. We are currently working with several international teams to extend Multilingual PubMed to German, Spanish, Portuguese, and Norwegian. According to the number of PubMed citations (see Table 1) for the main European languages, this feasibility study could be extrapolated as positive for other languages with fewer PubMed citations than French (ie, Spanish, Italian, Portuguese, and Norwegian) and one-third larger for German.

Several other improvements related to MeSH indexing in the search engine have not yet been implemented for PubMed citations; in particular, affiliation of MeSH qualifiers to MeSH supplementary concepts, indexing with MeSH concepts, and affiliation of MeSH qualifiers to MeSH concepts [26]. It will not be feasible to perform these tasks manually due to the size of the PubMed citations corpus in French (n=665,359). Therefore, the automatic indexing tool used in the CISMeF quality-controlled health gateway will need to be enhanced to be applied to the Multilingual PubMed-French search engine.

As stated in the Introduction, it is possible to manage multiple kinds of resources in the CISMeF information system. End users are now able to ask for mixed results: Web resources from CISMeF and PubMed citations from Multilingual PubMed. Also, an interesting avenue for future development of Multilingual PubMed is to integrate bibliographic data from other sources. A partnership with Elsevier-Masson and 2 small French companies has now been launched. Elsevier will provide additional metadata for MEDLINE/PubMed citations (abstracts in French in particular), which currently are not yet available in the MEDLINE/PubMed database. Furthermore, Elsevier-Masson will provide metadata on journals not included in PubMed, but included in other bibliographic/bibliometric databases (eg, Web of Science, EMBASE, and BIOSIS).

In addition to being more efficient than its competitors are, Multilingual PubMed is, in our opinion, more user friendly and provides more features. Nevertheless, there are still many interesting functionalities in PubMed (ie, related citations, history management) or in other tools [8] (graphical representation) that are not implemented in Multilingual PubMed. Future work may address these issues.

### Conclusion

This feasibility study was carried out to create a multilingual search engine to query monolingual PubMed subsets. It was successful for the French language and will be extended to the other main European languages. Bibliographical references from non-English publications can now be searched in a native language–friendly interface using multilingual data from the PubMed database and MeSH vocabulary.

Overall, this Multilingual PubMed tool could be of valuable interest for non–English-speaking health professionals who are unable to access PubMed.

## Abbreviations

CISMeF: Catalogue et Index des Sites Médicaux de langue Française
LIFO: last in–first out
MeSH: Medical Subject Headings
NLM: National Library of Medicine
PMID: PubMed identifier
TT: transliterated title
